# Detection of Host Cell Gene/HPV DNA Methylation Markers: A Promising Triage Approach for Cervical Cancer

**DOI:** 10.3389/fonc.2022.831949

**Published:** 2022-03-25

**Authors:** Lingyi Zhang, Wenxi Tan, Hongmei Yang, Songling Zhang, Yun Dai

**Affiliations:** ^1^ Laboratory of Cancer Precision Medicine, The First Hospital of Jilin University, Changchun, China; ^2^ Department of Gynecology and Obstetrics, The Second Hospital of Jilin University, Changchun, China; ^3^ Department of Critical Care Medicine, The First Hospital of Jilin University, Changchun, China; ^4^ Department of Obstetrics and Gynecology, The First Hospital of Jilin University, Changchun, China

**Keywords:** DNA methylation, host cell gene, high-risk human papillomavirus, cervical intraepithelial neoplasia, cervical cancer, triage

## Abstract

Cervical cancer is the most prevalent gynecologic malignancy, especially in women of low- and middle-income countries (LMICs). With a better understanding of the etiology and pathogenesis of cervical cancer, it has been well accepted that this type of cancer can be prevented and treated *via* early screening. Due to its higher sensitivity than cytology to identify precursor lesions of cervical cancer, detection of high-risk human papillomavirus (HR-HPV) DNA has been implemented as the primary screening approach. However, a high referral rate for colposcopy after HR-HPV DNA detection due to its low specificity in HR-HPV screening often leads to overtreatment and thus increases the healthcare burden. Emerging evidence has demonstrated that detection of host cell gene and/or HPV DNA methylation represents a promising approach for the early triage of cervical cancer in HR-HPV-positive women owing to its convenience and comparable performance to cytology, particularly in LMICs with limited healthcare resources. While numerous potential markers involving DNA methylation of host cell genes and the HPV genome have been identified thus far, it is crucial to define which genes or panels involving host and/or HPV are feasible and appropriate for large-scale screening and triage. An ideal approach for screening and triage of CIN/ICC requires high sensitivity and adequate specificity and is suitable for self-sampling and inexpensive to allow population-based screening, particularly in LMICs. In this review, we summarize the markers of host cell gene/HR-HPV DNA methylation and discuss their triage performance and feasibility for high-grade precancerous cervical intraepithelial neoplasia or worse (CIN2+ and CIN3+) in HR-HPV-positive women.

## Introduction

Invasive cervical cancer (ICC) represents the fourth most prevalent gynecologic cancer with an estimated 604,000 new cases, which causes approximately 342,000 deaths worldwide in 2020, especially in low- and middle-income countries (LMICs) (1). To achieve the ultimate goal of eliminating ICC globally (2), the WHO has called for innovative technologies to detect cervical precancerous lesions, namely, grade 2–3 cervical intraepithelial neoplasia (CIN2-3), as well as appropriate strategies to improve the coverage and acceptance rate of ICC screening (3). To this end, a high-quality screening approach is urgently needed to identify CIN lesions, most of which are curable before progression to ICC, and ICC occurrence as early as possible.

Many studies have revealed that DNA screening of high-risk human papillomavirus (HR-HPV) is superior to cervical cytology in the detection of CIN2 or worse (CIN2+) lesions (4–11), which has been used in many countries worldwide. However, the HR-HPV DNA test can also detect transient infections that are not clinically meaningful, leading to poor specificity for detecting CIN lesions and thus unnecessary colposcopy referrals, overtreatment, and adverse events, especially in young women. This is particularly important in LMICs where the healthcare resource is limited, in association with low accessibility and acceptance for cervical cancer screening. Thus, an effective triage approach with adequate cost-effectiveness is required to screen HR-HPV DNA-positive women (11). However, multiple triage assays, such as HPV E6/E7 mRNA test, cytology, and p16-INK4a/Ki-67 dual immunostaining (12–15), are not suitable for large-scale population-based screening due to their complicated procedures that require well-trained/experienced technicians as well as because they are better applied in physician-collected samples, rather than self-collected ones. Therefore, simple and inexpensive approaches with sufficient sensitivity and specificity are urgently needed for more precisely detecting cervical precancerous lesions and excluding transient HPV infections.

**Figure 1:**
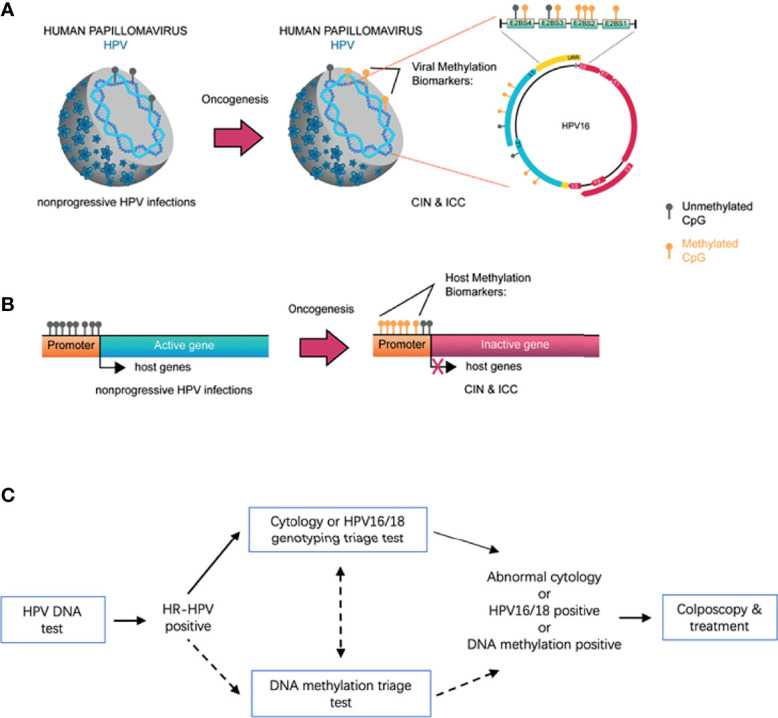
The mechanistic basis for detection of host gene and HPV DNA methylation for early screening of CIN/ICC. During persistent HR-HPV infection and cervical oncogenesis, hypermethylation of the host gene and viral genomic DNA is closely associated with the severity of CIN lesions and the risk of progression to ICC. With understanding the molecular mechanisms for HPV-mediated carcinogenesis, the positive correlation has been well established between CIN/ICC and hypermethylation of the CpG sites of HPV genomes (e.g., *L1*, *L2*, and *E2BS*) **(A)**, or between CIN/ICC and hypermethylation of the CpG sites of host cell gene promoters **(B)**. A number of studies have demonstrated that abnormal DNA methylations of host genes and/or HPVs, particularly in combination of multiple genes (i.e., gene panels), are capable of distinguishing non-progressive HPV infections from those associated with the potential to develop into ICC, therefore representing promising biomarkers for the triage of CIN lesions in HR-HPV infected women. Accordingly, a flowchart illustrates the potential process for the screening and triage of cervical cancer by integrating host cell gene/HPV DNA methylation detection in the future **(C)**.

During persistent HR-HPV infection and cervical oncogenesis, aberrant DNA methylation of human host cell genes or HPV genomic DNA is tightly associated with dysregulated functions of various tumor-suppressor genes (16–18). Moreover, DNA hypermethylation correlates with prolonged HR-HPV infection and is thus considered as a marker for severity of CIN lesions and risk of ICC (19). Emerging evidence supports a notion that detecting abnormal DNA methylation can distinguish the precancerous lesions from HPV infections that most likely do not develop to ICC, indicating its potential significance in the triage of patients who have CIN lesions (20) (**Figures 1A, B**), as shown in the flowchart for screening and triage of CINs (**Figure 1C**). In this review, we summarize the current status of detecting DNA methylation of host cell genes and HR-HPV for the triage of ICC precursor lesions in HR-HPV-positive women and discuss the advantages and limitations of diverse methylation markers identified thus far as well as their potential role in resolving controversial diagnosis of CIN2. To this end, a Medline search was performed for articles in English, using the MeSH terms “cervical” (Abstract) AND “cancer” OR “tumor” OR “neoplasia” OR “carcinoma” (Abstract) AND “methylation” (Abstract) AND “triage” (Text word) OR “screening” (Text word), which yielded a total of 286 papers. Among them, 180 articles were selected due to their close relationship with the content of this review article (e.g., HR-HPV-positive women as study subjects, self-sampling, population-based study, differentiation between CIN2+ and CIN3+, CIN2 diagnosis, etc.) while some additional original studies were also included due to their significant impacts on this field.

## Methylation-Mediated Cervical Carcinogenesis Driven by HPV Infection

According to their epidemiological relationship with the incidence of ICC, the HR-HPV family members are divided into HR-HPV, probable HR-HPV, and possible HR-HPV (**Table 1**) (21), while the latter two are identified only in about 3% cervical cancer and not sufficient to be included in population-based prevention programs due to lack of biological data (22). HPV16 and HPV18 are the most prevalent HR-HPVs (23). HR-HPVs contain six early genes (*E1*, *E2*, and *E4–7*), which facilitate the expression and replication of the viral genome, and two late genes (*L1* and *L2*) for capsid formation (24).

**Table 1:** Risk classification of HPVs in cervical cancer.

Category	Virus types
High risk HPV (HR-HPV)	HPV 16, 18, 31, 33, 35, 39, 45, 51, 52, 56, 58, 59
Probable HR-HPV	HPV 68
Possible HR-HPV	HPV 26, 53, 66, 67, 70, 73, 82

**Figure 2:**
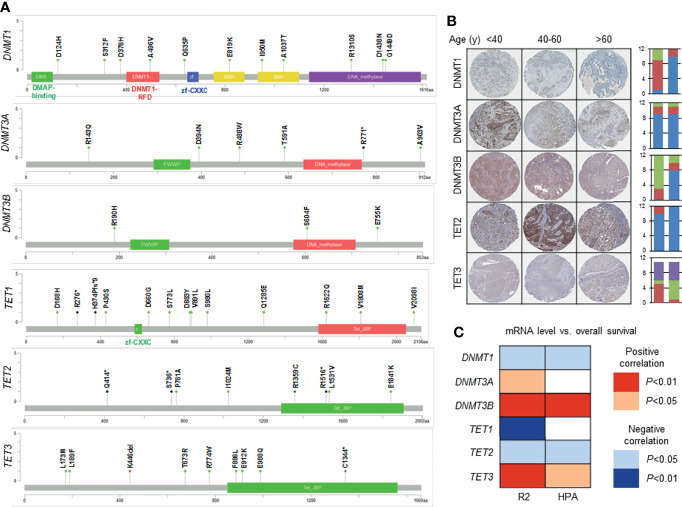
The alterations of DNA methylation-regulatory genes and their relationship with patient survival in ICC. The molecular alterations of DNA methylation modifiers at the genetic, transcriptomic, and protein levels, as well as their effects on the outcome of patients with ICC, are analyzed using various public available databases. **(A)** Genetic mutations (black—nonsense, green—missense, brown—in-frame deletion) of DNMTs and TETs in ICC, using the Cervical Squamous Cell Carcinoma dataset (TGCA, PanCancer Atlas; n = 294) on the cBioPortal for Cancer Genomics platform (www.cbioportal.org). **(B)** Immunohistochemical (IHC) staining of DNMTs and TETs in ICC, using the Cervical Cancer dataset on the Human Protein Atlas platform (www.proteinatlas.org). Representative images are shown. For each bar graph, the y-axis indicates the number of cases; the left bar indicates staining intensity (blue—strong, red—moderate, green—weak, purple—negative); the right bar indicates quantity (blue—>75%, red—25%~75%. green—<25%, purple—none). Note: the IHC data for TET1 are not available. **(C)** Relationship between mRNA levels of DNMTs or TETs and overall survival of ICC patients, using the Tumor Cervical Squamous Cell Carcinoma - TCGA dataset (TCGA ID: CESC; n = 303; DNMT1: high = 155, low = 148; DNMT3A: high = 270, low = 33; DNMT3B: high = 231, low = 72; TET1: high = 290, low = 13; TET2: high = 290, low = 13; TET3: high = 246, low = 57) on the R2: Genomics Analysis and Visualization Platform (http://r2.amc.nl) and the Cervical Cancer dataset (n = 291; DNMT1: high = 101, low = 190; DNMT3A: high = 204, low = 87; DNMT3B: high = 228, low = 63; TET1: high = 198, low = 93; TET2: high = 106, low = 185; TET3: high = 229, low = 62) on the Human Protein Atlas (HPA) platform (www.proteinatlas.org). Positive correlation = high mRNA levels correlate with poor outcome; Negative correlation = low mRNA levels correlate with poor outcome.

Infection with HR-HPV does not always result in ICC, while persistent HR-HPV infection that mediates cervical epithelial cell transformation is required for the development of precancerous lesions (CIN1–3) before progression to ICC. Therefore, the consequences of HR-HPV infection vary upon how cervical epithelial cells are exposed to HR-HPV, including transient, productive, and transforming infections (25). Transient infection is often not pathological due to spontaneous removal of HPV *via* local immunity. Productive infection is associated with productive CIN lesions (histologically corresponding to CIN1 or CIN2) that could spontaneously regress in 1–2 years, likely due to viral clearance. In contrast, transforming infections are associated with some CIN2 and most CIN3 lesions that are highly heterogeneous in regard of lesion regression versus oncogenesis, with a varied expression of *E6* and *E7* as well as patient survival (21). During carcinogenesis, the integration of HPV DNA to the host genome is accompanied by the downregulation of *E2* but upregulation of oncogenic *E6* and *E7* (25–27). Moreover, the modified E2-binding sites (E2BS) in the upstream regulatory region (URR) or altered *E2* expression may also be involved (28–30). For example, methylation of E2BS in the HPV16 enhancer region promotes disease progression (16, 17). However, the methylation status of a specific locus in the *E6* promoter and enhancer is irrelevant to the role of HPV16 in the development of cervical precancerous lesions (31). *E6* and *E7* can regulate both cellular and viral gene expressions by modifying DNA methylation. *E6* upregulates the expression of the DNA methyltransferase DNMT1 by suppressing *TP53* (32), while *E7* directly binds to and activates DNMT1 (33). Overexpression of *E6*/*E7* oncoproteins drives uncontrolled cell proliferation, impaired apoptosis, and enhanced DNA damage repair by regulating related proteins, such as p53 and pRb (34). It also leads to chromosomal instability, accumulation of oncogenic alterations involving host cell genes, hypermethylation of tumor-suppressor gene promoters that silences target genes, and therefore malignant transformation of HR-HPV-infected cells (18, 34–36). DNA methylation refers to covalent transfer of a methyl group to the C-5 position to form 5-methylcytosine (5mC). DNA modification by methyl groups leads to translocation of transcription factors and thus alterations of gene expression by changing chromatin structure and DNA topology (37, 38). In general, hypermethylation of the CpG-rich region in gene promoters (CpG islands) suppresses transcription of target genes (21). Mechanistically, DNA methylation (e.g., 5mC) is reciprocally regulated by DNA methyltransferases (e.g., DNMT1, DNMT3A, and DNMT3B) as epigenetic writer and ten-eleven translocation (TET) methylcytosine dioxygenases (e.g., TET1-3) as eraser. To understand the potential role of DNA methylation dysregulation in ICC, we performed genome-wide analyses on the alterations of DNA methylation-regulatory genes (i.e., DNMTs and TETs) using the publicly available genomic, transcriptomic, and protein databases, including the Cervical Squamous Cell Carcinoma dataset (TGCA, PanCancer Atlas) on the cBioPortal for Cancer Genomics platform (www.cbioportal.org), the Tumor Cervical Squamous Cell Carcinoma - TCGA dataset (TCGA ID: CESC) on the R2: Genomics Analysis and Visualization Platform (http://r2.amc.nl), and the Cervical Cancer dataset on the Human Protein Atlas platform (www.proteinatlas.org), respectively. Of note, mutations of these DNA methylation-modifying genes are relatively rare (**Figure 2A**), including *DNMT1* (5%), *DNMT3A* (2.7%), *DNMT3B* (4%), *TET1* (4%), *TET2* (2%), and *TET3* (2.4%); however, DNMT3A and TET2 are relatively high at the protein level but vary with different ages, while the levels of DNMT1, DNMT3B, and TET3 are moderate or relatively low (**Figure 2B**); and the expression of these genes correlates, either positively (for *DNMT3A*, *DNMT3B*, and *TET3*) or negatively (for *DNMT1*, *TET1*, and *TET2*), with the overall survival of ICC patients (**Figure 2C**). These observations suggest that the abnormalities of host cell DNA methylation may be associated with dysregulation of its regulatory machinery involving DNMTs and TETs, which in turn contribute to disease progression and outcome of ICC patients.

In addition to host cell DNA methylation, HPV DNA can also be methylated during malignant transformation. Viral genome methylation has either negative or positive effects on viral gene transcription. The HPV genome has highly condensed and conserved CpG sites (39). Altering CpG site methylation switches HPV infection from productive to transforming type (29). During carcinogenesis, HR-HPV-mediated hypermethylation is associated with the severity of cervical lesions, especially ICC (21, 40). Consistently, CIN2-3 lesions in patients with long-term (≥5 years), rather than short-term, HR-HPV infection exhibit cancer-like hypermethylation and abnormal chromatin features (40). Due to its specificity for CIN lesions and ICC pathogenesis, HPV DNA hypermethylation has attracted considerable attention in CIN/ICC screening and has been widely investigated in cervical histological and cytological specimens.

## Markers of Host Cell Gene Methylation

An ideal biomarker involving DNA methylation is able to distinguish the majority (>90%) of cancer cases from non-malignant ones (41). A great number of studies have attempted to identify such markers for CIN/ICC screening. A meta-analysis on the methylation of 68 different genes from 51 studies has revealed high heterogeneity of the published data, which makes it difficult to identify appropriate methylation markers for screening cervical cancer (20, 36). Nonetheless, a number of studies have demonstrated the panels of methylated genes and non-coding RNAs for the screening and triage of CIN/ICC, of which the studies with a relatively large sample size of HR-HPV-positive women are summarized in **Table 2**. Among many genes investigated in these studies, some representative examples that have been relatively well studied are discussed as follows.

**Table 2:** Comparison of host gene methylation markers alone or in combination for detecting CIN2+/CIN3+ in HR-HPV-positive women.

Gene/gene panel	Tissue type	Sample number	CIN2+		CIN3+		Ref
			Sensitivity (%)	Specificity (%)	Sensitivity (%)	Specificity (%)	
FAM19A4	Scrapes	218	69.2	69.9	75.8	67.0	(42)
FAM19A4	Scrapes	508	57.8	74.1	75.6	71.1	(43)
FAM19A4	Vaginal lavage	450	44.4	82.8	65.3	81.3	(44)
JAM3	Scrapes	128	63.0	90.0	82.0	88.0	(45)
C13ORF18	Scrapes	128	49.0	92.0	65.0	91.0	(45)
EPB41L3	Scrapes	128	67.0	57.0	88.0	61.0	(45)
TERT	Scrapes	128	69.0	62.0	76.0	60.0	(45)
ZNF582	Scrapes	230	NA	NA	83.0	71.0	(46)
PTPRR	Scrapes	230	NA	NA	92.0	49.0	(46)
PAX1	Scrapes	230,55	NA	NA	46.0,60.0	86.0,100.0	(46, 47)
SOX1	Scrapes	230,55	NA	NA	62.0,63.0	76.0,74.0	(46, 47)
POU4F3	Scrapes	100	NA	NA	88.0	82.0	(48)
POU4F3	Scrapes	55	NA	NA	74.0	89.0	(47)
CADM1/MAL	FFPE	261	NA	NA	70.0	78.0	(49)
CADM1/MAL	Scrapes	236	NA	NA	60.5~100	83.3	(50)
FAM19A4/miR124-2	Scrapes	2384	33.3~61.1	71.1~80.3	75.0~78.2	71.1~80.3	(51)
CCNA1/C13ORF18	Scrapes	89	37.0	96.0	NA	NA	(52)
C13ORF18/JAM3/ EPB41L3/TERT	Scrapes	107	65.0	NA	82.0	NA	(53)
C13ORF18/JAM3/ ANKRD18CP	Scrapes	215	74.0	76.0	NA	NA	(54)
C13ORF18/EPB41L3/ JAM3	Scrapes	235	80.0	66.0	95.0	64.0	(55)
ANKRD18CP/ C13ORF18/JAM3	Scrapes	235	60.0	68.0	68.0	67.0	(55)
SOX1/ZSCAN1	Scrapes	235	63.0	84.0	79.0	81.0	(55)
DLX1/ITGA4/RXFP3/ SOX17/ZNF671	Scrapes	217	56.0	88.7	76.2	82.9	(56)
AJAP1/MAG12/POU4F3	Scrapes	100	NA	NA	73.0	97.0	(57)
AJAP1/EDN3/EPO/ MAGI2/SOX17	Scrapes	100	NA	NA	73.0	98.0	(57)
DAPK1/RARB/TWIST1	Biopsy	319	NA	NA	52.0	95.0	(58)
ASTN1/DLX1/ITGA4/ RXFP3/SOX17/ZNF671	Scrapes	189	NA	NA	67.4	76.0	(59)
EPB41L3/SOX1/DCC	Scrapes	167	NA	NA	70.0	91.0	(60)
EPB41L3/SOX1	Scrapes	167	NA	NA	76.0	87.0	(60)
EPB41L3/DCC	Scrapes	167	NA	NA	76.0	86.0	(60)
SOX1/DCC	Scrapes	167	NA	NA	75.0	84.0	(60)
SOX1/PAX1/LMX1A/ NKX6-1	Scrapes	185	NA	NA	88.0	82.0	(61)

NA, not available.

### *CADM1* and *MAL*

*CADM1* (also known as *TSLC1*) is a tumor-suppressor gene that encodes an intracellular adhesion protein of the immunoglobulin superfamily (62). Silencing of *CADM1*, due to its promoter hypermethylation, is common and associated with the severity of ICC (63). Functionally, CADM1 is involved in anchorage-independent growth of ICC cells (64). The frequency and density of methylation at the *CADM1* promoter positively correlate with the grade of CIN lesions. Of note, abnormal methylation of the *CADM1* promoter in cervical cytological biopsies could be detected 7 years before the diagnosis of ICC (63).

In chromosomal and transcriptional profiles of ICC, the *MAL* gene is downregulated in ICC samples compared to normal ones (65). *MAL* encodes T lymphocyte maturation-associated protein involving apical membrane transport and secretion (66, 67). Silencing of *MAL via* its promoter methylation contributes to the transformation and oncogenesis of HPV-infected cervical cells, suggesting *MAL* methylation as a diagnostic biomarker for squamous cell carcinoma (SCC) and adenocarcinoma (AdCA), as well as their precancerous lesions (68). However, another study has shown that *MAL* methylation fails to discriminate different grades of cervical lesions, possibly due to different methods and samples used to detect this methylation (69).

A panel combining *CADM1* and *MAL* methylation can improve the specificity of screening cervical scraping samples and identify 90% of HR-HPV-positive women with CIN3 (49). Moreover, a prospective cohort study has investigated the triage value of a *CADM1/MAL*-specific methylation panel, demonstrating its performance comparable to cytology alone or in combination with HPV16/18 genotyping in distinguishing CIN3+, without missing any SCC/AdCA cases (50). However, a potential bias in favor of cytology cannot be ruled out because the women enrolled in this study are primarily referred to abnormal cytology. Notably, *CADM1/MAL* methylation is higher in CIN2/3 (5.3- to 6.2-fold) and carcinomas (143.5- to 454.9-fold) than those in CIN1 or normal cervical tissues; their methylation levels further increase (11.5- and 13.6-fold) in CIN2/3 with long-term HR-HPV infection (>5 years) (40). On the other hand, *CADM1/MAL* methylation is lesion-specific and associated with CIN severity, likely to indicate the worst lesions in women with multiple cervical lesions, particularly CIN3+ (70). Therefore, the panel combining *CADM1* and *MAL* methylations may represent a promising triage approach for HR-HPV-positive women.

### *FAM19A4* and *Hsa-miR-124*

Hypermethylation of *Hsa-miR-124*, a well-recognized epigenetic silencer, has been observed in many types of cancer, including lung, breast, hepatocellular, gastric, and colon cancer, as well as leukemia (71–74). *Hsa-miR-124* methylation increases in cervical scrapes and tissue specimens of women with ICC and its precancerous lesions, thus representing a common event functionally involved in oncogenesis of ICC (75).

*FAM19A4* methylation has been identified as a promising biomarker for cervical oncogenesis *via* genome-wide DNA screening (76). In a large cohort of HR-HPV-positive women with high quality of cytology reading in Netherlands, *FAM19A4* methylation assay could detect advanced CIN lesions and cervical cancer, with sensitivity and specificity of 75.8% and 67.0% for CIN3+, 69.2% and 69.9% for CIN2+, respectively (42). Moreover, the *FAM19A4* methylation test displayed better clinical performance than cytology for most advanced lesions. Although its triage performance varies with age, *FAM19A4* methylation is sensitive for detecting long-term CIN3 lesions and ICC (43). In HR-HPV-positive women with age >30 years, *FAM19A4* methylation for detecting CIN3 exhibits similar sensitivity (88.3% versus 85.0%) and higher specificity (62.1% versus 47.6%) when compared with cytology. However, in women with age <30 years, its sensitivity is lower (50% versus 86.7%) than cytology, while the specificity is significantly higher (81.7% versus 52.4%). One explanation for such inconsistency between women with different ages is that younger women are more likely to have early CIN lesions and relatively short-term HR-HPV infection (77, 78). These findings indicate *FAM19A4* methylation as an objective molecular triage marker for HR-HPV-positive women. This triage marker may be particularly useful in LMICs with poor cytology infrastructure, because it is not crucial to detect all precancerous lesions in the first screen as most of these lesions progress considerably slowly or even regress (79). While the age for ending the cervical cancer screening remains controversial, DNA methylation may offer more accurate screening-exit strategies as its alterations vary with age (80). The triage capacity of *FAM19A4/miR-124-2* methylation in HPV-positive women with age >30 years, using the samples from the POBASCAM trial, has revealed 1.7% and 2.4% of 14-year cumulative ICC incidence in baseline methylation-negative and cytology-negative women, respectively (81). Compared to a negative cytology result, a negative result of the *FAM19A4/miR-124-2* methylation test has similar capability to exclude the long-term risk of CIN3+ in HPV-positive women with age >30 years (82). In a multicenter prospective study, the triage capacity of combining *FAM19A4/miR-124-2* methylation with HPV16/18 genotyping displayed high sensitivity (93.1% and 98.2%) and moderate specificity (49.4% and 46.3%) in women with either CIN3+ colposcopy results or age >30 years, better than either methylation test or HPV genotyping alone (83).

High sensitivity and specificity of the *FAM19A4/miR-124-2* methylation test have been further validated for screening CIN/ICC in gynecologic outpatients in several other studies. In a large cohort of patients from 25 countries worldwide, the assessment of *FAM19A4/miR-124-2* methylation is able to exclude the possibility of ICC by negative results, and its performance is not affected by sample types and geographical regions (84). The QIAsure methylation test used in this study to measure *FAM19A4/miR124‐2* methylation has been further demonstrated to have high intra- and inter-laboratory agreement in another international study, confirming its accuracy and reproducibility, which warrant the robustness of this assay (85). Notably, the level of *FAM19A4/miR-124-2* methylation in ICC is independent of histology type, geographical area, sample type, and HPV genotype (84). This finding has further been verified in a multicenter retrospective study (named VALID-SCREEN) (51). Moreover, this study has also revealed that the *FAM19A4/miR-124-2* methylation test has an overall sensitivity of 77.2% and specificity of 78.3% for CIN3. Such high sensitivity and NPV for CIN3+ could be helpful to reduce the colposcopy referral rate and prolong the time to reexamination in clinical practice. Therefore, the FAM19A4/miR124-2 methylation test may represent a milestone for practical implementation of DNA methylation in cervical cancer screening.

### Other Genes

In addition to the promising methylation markers described above, many other potential genes and panels have also been investigated in screening cervical tissue specimens or exfoliated cells.

The tumor-suppressor gene *C13ORF18* encodes a phosphatase inhibitor, the reactivation of which inhibits tumor cell growth and induces apoptosis (86). *C13ORF18* has been investigated virtually all in combination with other genes thus far. For example, methylation at the promoters of *CCNA1* and *C13ORF18* is associated with the severity of cervical lesions (52). Methylation of both genes displays high specificity for CIN2 or higher (97% and 100%, respectively), while the sensitivity for these lesions is low (37%) (52). A combination of primary HR-HPV testing with an assay using a methylation panel of *JAM3*, *EPB41L3*, *TERT*, and *C13ORF18* can improve the capacity to detect CIN3+, when compared to the triage test combining HR-HPV with cytology, though in a hypothetical scenario analysis (53). A genomic methylation test of nine genes (*ZSCAN1*, *ST6GALNAC5*, *ANKRD18CP*, *PAX2*, *CDH6*, *GFRA1*, *GATA4*, *KCNIP4*, and *LHX8*) displays significantly different methylation patterns between CIN2/3 lesions and normal cervical samples. The combination panels of *C13ORF18/JAM3/ANKRD18CP* exhibited comparable sensitivity and specificity to cytology for CIN2+ in HR-HPV-positive scrapings (54). Unlike most of single-gene methylation markers that require a cutoff value to reach high specificity, no cutoff value is required for these methylation panels involving multiple genes. In a cohort of HR-HPV-positive patients, the values of three combinations involving six genes (*JAM3*, *EPB41L3*, *C13ORF18*, *ANKRD18CP*, *ZSCAN1*, and *SOX1*) for diagnosing CIN3+ have been evaluated in women who underwent colposcopy. While all three methylation panels (*EPB41L3/JAM3*, *ANKRD18CP/C13ORF18/JAM3*, and *SOX1/ZSCAN1*) exhibited high NPVs with sensitivities in a range of 68% to 95%, the *SOX1/ZSCAN1* panel has higher specificity (84%) than the other two (55). Therefore, the methylation panels of multiple genes, rather than a single-gene methylation test, may provide a promising triage approach for cervical cancer screening.

*EPB41L3* was initially identified as a tumor suppressor that is downregulated in lung carcinoma (87). Loss of its tumor suppressor activity has also been observed in breast cancer (88), prostate cancer (89), and meningiomas (90). *EPB41L3* downregulation is most likely due to its promoter hypermethylation (91). An *EPB41L3* methylation assay has been used to detect ICC and its precancerous lesions with promising performance, either alone or in combination with other human or viral genes, to triage HR-HPV-positive women and identify those with a risk of cervical oncogenesis (69). *EPB41L3*/*ANKRD18CP* methylation has lower sensitivity to detect CIN2+ in younger women than those with age >30 years (55). The lower methylation rate for younger women (<30 years) may be due to their relatively shorter period of HPV infection. Similar phenomena have been observed when another methylation signature involving five genes (DLX1, ITGA4, RXFP3, SOX17, and ZNF671) specific for CIN3+ was used (56).

*ZNF582* is involved in DNA damage response, cell proliferation, cell-cycle regulation, and neoplastic transformation (92). Several robust studies have demonstrated that *ZNF582 combined with other genes* (*ZNF582/PTPRR/PAX1*/*SOX1*, *ADCY8/CDH8/ZNF582*) have excellent triage performance for women with cervical abnormal cytology (46, 93), while PAX1 methylation (PAX1^m^) alone has also shown a triage performance comparable to cytology and better accuracy, with higher specificity than HPV16/18 as the triage tool for CIN3+ in HR-HPV-positive women (94). In addition, a commercialized diagnostic test of *ZNF582* methylation is undergoing development (95).

*POU4F3* has diverse biological functions, including transcriptional regulation, cellular differentiation, and metabolic processes (57). Hypermethylation of *POU4F3* suggests its potential role as a tumor suppressor in ICC (48). A retrospective case–control study has revealed that *POU4F3* methylation exhibits good triage efficacy for CIN3+ in HR-HPV-positive women, with sensitivity of 74% and specificity of 89%, respectively (47).

Hypermethylation of *DAPK1*, *RARB*, or *TWIST1* is able to identify histologically confirmed CIN3+, with specificity of 95% and relatively low sensitivity (60%) in a community-based study involving 2,609 women, which has thus been considered to have the screening performance superior to HR-HPV detection or abnormal cytology. The triage capacity of gene methylation (e.g., *DAPK1* and *RARB*) for CIN3+ is comparable between African and non-African populations, suggesting potential use of these gene methylations in different populations (58).

Several methylation panels involving multiple genes have also been widely investigated. The triage efficacy of GynTect^®^ (Oncgnostics GmbH, Jena, Germany), a methylation-specific real-time PCR assay targeting *ASTN1*, *DLX1*, *ITGA4*, *RXFP3*, *SOX17*, and *ZNF671*, could identify all cancer cases with an overall sensitivity of 67.7% for detecting CIN3+, particularly in women with age ≥30 years probably due to their long-term HR-HPV infection (59). In a comparison between two commercial DNA methylation-based diagnostic assays, GynTect^®^ seems preferable due to its higher specificity for CIN2+ or CIN3+ than the QIAsure assay (96).

In an early study, methylation abnormalities of six genes (*RRAD*, *SFRP1*, *MT1G*, *NMES1*, *SPARC*, and *TFPI2*) have been observed in a subset of ICC samples but not in control samples (97). Methylation of twelve candidate genes (*ADCYAP1*, *ASCL1*, *ATP10*, *CADM1*, *DCC*, *DBC1*, *HS3ST2*, *MOS*, *MYOD1*, *SOX1*, *SOX17*, and *TMEFF2*) is significantly increased in CIN3+ but not lesions with grade <CIN2. Among them, methylation of seven genes (*ADCYAP1*, *DCC*, *EPB41L3*, *HS3ST2*, *miR-124*, *MOS*, and *SOX1*) has high accuracy for detecting CIN3+, despite the differences in methodology (60). A meta-analysis has validated that increased methylation at the promoters of several genes (*CADM1*, *MAL*, *miR-124-2*, *FAM19A4*, *POU4F3*, *EPB41L3*, *PAX1*, and *SOX1*) and HPV16 *L1/L2* correlates to CIN lesion, with good performance in the triage of advanced CIN (20). While *PEG3* methylation is associated with advanced CIN lesions (98), its efficacy for screening CIN/ICC needs to be further investigated. Because 3q gain is common in ICC, methylation of three genes (*GHSR*, *SST*, and *ZIC1*) is associated with 3q gain as well as the severity of CIN (99).

Among various histological types of ICC, SCC accounts for 80% of cases and thus represents the most common type, followed by AdCA (10%–20%), while other types include adenosquamous carcinoma and several rare ones (21). In this context, several methylation markers have been investigated in a histotype-dependent manner (100–106). For example, *CADM1* is prevalently associated with cervical lesions in SCC than AdCA (64), while methylation of *APC*, *TIMP3*, and *RASSF1A* may distinguish AdCA from SCC (100). Methylation of *MAL* and *FAM19A4* promoters is significantly increased in both SCC and AdCA compared to precancerous lesions (68, 76). Other genes (e.g., *CDH1, DAPK1, EPB41L3, PAX1, PRDM14*, and *TERT*) could also be methylated in SCC and adenocarcinoma (21). However, methylation of *FAM19A4/miR-124-2* is common in ICC, including its rare histotypes (84).

Together, methylation of numerous host cell genes, either alone or in combination, have been considered powerful biomarkers to predict the progression of cervical cancer via the triage of HR-HPV positive women.

## HR-HPV DNA Methylation Markers

Plenty of studies have focused on methylation changes in the HPV genome to identify specific markers for distinguishing CIN from ICC (**Table 3**). By understanding the molecular mechanism for HPV-mediated carcinogenesis, a link between CIN/ICC and methylation of the CpG sites of HPV *L1*, *L2*, *E2-E4*, *E5*, and *URR* has been well established. Particularly, a positive correlation between the level of *L1* methylation and CIN2+ lesions appears to be consistent among multiple studies (107, 108, 116–120). However, the relationship between *URR* methylation and the severity of precancerous lesions remains controversial (17), likely due to the nature of the samples, differences in the CpG sites analyzed, and the diverse methods used.

**Table 3:** Comparison of HPV DNA methylation markers for detecting CIN2+/CIN3+.

Gene/Gene panel	Tissue type	Sample number	CIN2+		CIN3+		AUC	Ref
			Sensitivity(%)	Specificity(%)	Sensitivity(%)	Specificity(%)		
HPV18,31,45 genomes	Cytology	188	NA	NA	NA	NA	0.85/0.81/0.98	(107)
HPV16 genome	Exfoliated cell	273	91.0	60.0	NA	NA	0.82	(108)
HPV16 L1	Cytology	145	NA	NA	75.7	77.5	0.85	(109)
HPV16 L1/LCR	Cytology	77	NA	NA	85.7	78.4	0.80	(110)
HR-HPV 16/18/31/33/35/39/45/51/52/56/58/59 L1/L2	Cytology	659	NA	NA	80.0	65.6	0.46	(111)
EPB41L3/HPV16/18/31	Cytology	1493	90.0	36.0	NA	NA	0.80	(112)
EPB41L3/HPV16/18/31/33	Cytology	1493	90.0	49.0	NA	NA	0.82	(113)
EPB41L3/HPV16/18/31/33	Cytology	341	74.0	65.0	NA	NA	0.78	(114)
EPB41L3/HPV16/18/31/33	Cytology	257	75.7	44.0	93.2	41.8	0.81/0.85	(115)
EPB41L3/HPV16/18/31/33	Exfoliated cell	316	62.0	73.0	70.3	76.6	0.75/0.81	(79)

NA, not available; AUC, area under the curve.

Hypermethylation at *L1*, *L2*, and *E2–4* CpG sites increases the risk of CIN3 (50-fold) compared to hypomethylation. Moreover, an increase in their methylation levels is associated with high-grade squamous intraepithelial lesion (HSIL) and the risk of advancing to CIN2+ in HPV16-positive women (116). The diagnostic value of HPV genome methylation has also been demonstrated in women with age >28 years, of which CpG methylation of *L1* and *L2* is particularly higher in pre-diagnostic CIN2+ samples (median time to diagnosis = 3 years) than control samples (108). The performance of HPV16 *L1* CpG 6457 methylation to detect CIN2+ (sensitivity of 91.1% and specificity of 60.2%) (108) is comparable to that for p16-INK4a immunostaining (sensitivity of 92.6% and specificity of 63.2%) (121). However, it remains to be determined whether these viral methylation changes could predict CIN2+. The performance of HPV16 *L1*, *L2* DNA methylation is consistent in different populations, suggesting a potential for expanding its application (116, 122). The average methylation level of twelve HPV16 *L1* CpG loci is significantly higher in CIN3+ than in CIN1 or CIN2. However, the methylation of its loci 5,611 and 7,145 is more accurate to predict disease status than the average methylation level of 12 CpG sites, suggesting that the methylation test of a small number of CpG sites may represent a cost-effective option (109). Combining the HPV16 methylation test of six CpG sites (5,602, 6,650, 7,034, 7,461, 31, and 37) with E6 oncoprotein detection improves long-term risk stratification *via* triaging HPV16-positive women (110). Moreover, when combining with the E6 oncoprotein detection, the methylation test of either a single site or a panel covering multiple sites displays better predictive values than cytology.

Most studies on HR-HPV methylation and its relationship with premalignant cervical lesions have been focused on CpG sites of the HPV 16 genome (16). Approximately 40% of women with HPV16-positive cervical exfoliated cells have CIN2+ lesions (123). In the HPV16-negative women, the majority of CIN3+ cases are positive for other HR-HPV genotypes at baseline. Thus, the integration of the HPV methylation assay into the CIN/ICC screening may reduce the possibility of missing CIN3+, at least to a certain extent (110). In this context, multiple HPV DNA methylation assays can be used to improve HPV screening and genotyping for ICC (124). The whole-genome methylation analysis of HPV18/31/35 has indicated that methylation of these HR-HPV forms is common in CIN3 patients and may help determine which one is the causal infection (107). Moreover, this analysis has also revealed a high discrepancy in methylation of *L1*, *L2*, and *E2* genes between CIN and ICC or CIN3 and SCC, while the methylation levels of *URR*, *E1*, *E6*, and *E7* are considerably low. Methylation of HPV16/18/51 CpG sites is significantly higher in *L1* than in URR for all three HPV forms, while the degree of methylation positively correlates with the severity of cervical neoplasia (125).

A population-based screening by co-testing cytology and HPV has indicated a clear link between increased methylation and CIN3/AIS cross all twelve HR-HPV forms, suggesting that HPV DNA methylation is a common phenomenon in transforming HPV infection. DNA methylation involving HR-HPV forms 5, 10, or 12, either alone or in combination with HPV16/18 genotyping, has a higher capability for risk assessment than cytology (111). Patients with negative results of the combination tests involving HR-HPV forms 10 and 12 also display a lower risk than cytology. However, they are currently used only in the high resource countries due to their cost.

## The Combination of the Host Cell Gene and HPV DNA Methylation

A test (named S4 classifier) combining the host gene *EPB41L3* with *HPV16*-*L1/L2*, *HPV18*-*L2*, and *HPV31-L1* to predict CIN2/3 increases the PPV with minimal loss of sensitivity in HR-HPV-positive women with abnormal cytology (112). Then, the S5 classifier upgraded from S4 by adding HPV33 significantly improves the ability to estimate early risk, with sensitivity of 90% and specificity of 49% (cut point = 0.8) for CIN2/3 in a colposcopy-referred population in Western Europe (113). Due to the limited number of the cases that could be selected from the colposcopy-referred population, the performance of S5 has been further assessed in exfoliated cervical samples from a population-based routine screening. The results have shown better sensitivity of S4 (cut point = 0.5; 69% and 74% for CIN2+ and CIN3+) or S5 (cut point = 0.8; 74% and 84% for CIN2+ and CIN3+) than HPV16/18 genotyping (54% and 58% for CIN2+ and CIN3+), with similar specificity between S5 and HPV16/18 genotyping in HPV-positive women (114). Furthermore, the performance of S5 has been evaluated in a population-based randomized controlled trial named HPV for Cervical Cancer Screening (HPV FOCAL) in North America, indicating that S5 detects a greater percentage of advanced CIN lesions (cutoff = 0.91; sensitivity = 75.7% and 93.2%, specificity = 44% and 41.8% for CIN2/3 or CIN, respectively) than other approaches (e.g., abnormal cytology and HPV16/18 genotyping) (115). These observations suggest that the S5 classifier could help identify women with a high short-term risk of progression to ICC who need immediate treatment. Another study for evaluating the performance of the S5 classifier to predict CIN2+ in HR-HPV-infected women referred for colposcopy has demonstrated that this classifier has high AUC (area under the curve) values in distinguishing women with CIN2+ from those with lesion grade **<** CIN2 (AUC = 0.75, 95% CI: 0.69–0.82) and for CIN3+ (AUC = 0.81, 95% CI: 0.74–0.89). As consequence, the S5 classifier could reduce colposcopy referrals by 30%–50% without affecting sensitivity for CIN2+ and CIN3+, therefore significantly improving cost-effectiveness to allow identification of women with a true risk of ICC (79).

Together, the assays for host cell gene and HR-HPV DNA methylation alone and particularly in combination represent a promising triage approach with high sensitivity, specificity, and PPV for screening premalignant cervical diseases in HR-HPV-positive women. Although most evidence has been obtained from cross-sectional or retrospective studies with a limited sample size thus far, such an approach warrants further investigation in prospective screening and intervention studies, especially to optimize host cell gene and HR-HPV DNA methylation markers.

## The Potential Role of DNA Methylation Markers in Resolving Equivocal Diagnosis of CIN2

CIN2 is an equivocal diagnosis and thus considered as the least reproducible histopathologic type (126, 127), whereas approximately 40% of CIN2 lesions are manageable and only 5% would progress to ICC, compared to 33% and 12% for CIN3, respectively (128). Moreover, <1.5% of untreated patients with HSIL would progress to ICC within 24 months (129). A population-based study has shown that no less than 40% of young women (age ≤21 years) with histologically confirmed CIN2 experience spontaneous lesion regression during conservative follow-up with a median period longer than a year (130). Similarly, several studies have also revealed a high regression rate of histologically confirmed CIN2 lesions in young women (131, 132). Considering such a high regression rate, together with the risk of treatment-related adverse pregnancy outcomes (e.g., premature rupture of membranes or preterm labor) (133, 134), immediate treatment for biopsy-confirmed CIN2 lesions needs to be cautious, especially in young women who have fertility considerations (130, 135).

CIN2 represents a group of mixed lesions, including those from HPV infections (productive infections) to cancer precursors (transforming infections) as well as some early-transforming infections with insignificant risk of short-term progression to ICC (135, 136). Whereas the treatment options for CIN2 lesions vary, caution needs to be taken due to the fact that CIN2 lesions with productive infection and transforming infection cannot be distinguished morphologically (21). However, early-transforming infections could be distinguished from advanced transforming lesions based on their different degrees of genetic and epigenetic alterations. The HR-HPV-positive scrapings from women with CIN2/3 display heterogeneous DNA methylation patterns (involving *ANKRD18CP*, *C13ORF18*, *EPB41L3*, *JAM3*, *SOX1*, *ZSCAN1*, *GHSR*, *SST*, *ZIC1*, *FAM19A4*, *PHACTR3*, and *PRDM14* genes). A study has revealed that three-quarters of CIN3 samples and half of CIN2 samples display a cancer-like hypermethylation pattern, suggesting a high risk of progression to ICC (137). Another study using methylation tests of three genes (*SOX1*, *PAX1*, and *LMX1A*) has shown that the percentage of methylation reference is significantly higher in CIN3+ than those for the normal cervix and CIN1 or CIN2 (61). Notably, comparable results between physician- and self-collected samples have been obtained when similar genes (*PAX1, SOX1*, and *ZNF582*) were used to detect CIN3+ (138). These observations raise a possibility to distinguish productive infections from transforming ones *via* examining the molecular alterations associated with transformation from viral infection to ICC. In this context, patients with high methylation levels, associated with advanced CIN lesions, should receive immediate treatment, while those with low methylation levels, reflecting low short-term progression risk of ICC, only need close follow-up.

Women with CIN2+ lesions and positive *CADM1/MAL* methylation are often older than those with methylation-negative CIN2+, in association with the fact that CIN2+ lesions in young women could spontaneously regress and thus belong to early CIN lesion (70). A methylation panel of *ANKRD18CP* and *EPB41L3* genes has relatively low sensitivity to detect CIN2+ in women with age <30 years (55), consistent with other methylation tests involving *DLX1*, *ITGA4*, *RXFP3*, *SOX17*, and *ZNF671* genes (56), or *FAM19A4* (43), while low methylation rates have been observed in young women with CIN. In this context, young women are considered to have a shorter period of HPV infection and thus a lower methylation rate of target genes. In contrast, older women (age >30 years) with CIN2+ lesions more often have persistent HPV infection, in association with higher methylation rates and more advanced lesions (55). However, large longitudinal and multi-population studies are required to define the nature of CIN2 lesions with or without DNA methylation and to validate whether CIN2 lesions with a negative or low methylation level are less aggressive than those with a positive or high methylation level.

## Incorporation of Self-Sampling Into DNA Methylation-Based Screening and Triage

Even in Western countries (e.g., Netherlands, UK, and US), there are approximately 30% of women who have not participated in annual cervical screening programs, while these women may however face a high risk of development to CIN and ICC (139–142). Moreover, more than 50% of ICC patients are diagnosed *via* various procedures other than population-based screening programs. Older non-attendees have a relatively higher risk of CIN lesions than younger ones, likely due to their poor screening history (143). This problem is even more severe in LMICs. Therefore, the development of a simple, inexpensive, and acceptable screening approach is crucial to increasing the participation rate of screening and thus reducing the incidence of ICC. In this case, the introduction of self-sampling HR-HPV tests could reduce the percentage of non-attendees (144–147). A systematic review and meta-analysis has demonstrated that self-sampling HR-HPV tests have comparable accuracy to those using physician-collected cervical scrapings (144, 148).

The DNA methylation analysis of *C13ORF18*, *JAM3*, *EPB41L3*, and *TERT* using a self-sampling lavage device has the diagnostic performance non-inferior to cytomorphology and the HR-HPV tests (149). The triage performance of the DNA methylation test using these four genes is also comparable between self- and physician-collected samples (45). Consistently, another methylation analysis of similar genes has also shown similar triage capacity when the samples were collected, transported, and stored under dry conditions, an approach more convenient than cervicovaginal lavage (150). A self-sampling *MAL/miR-124-2* assay for CIN3+ and CIN2+ exhibits higher sensitivity than HPV16/18 genotyping (151). A randomized controlled trial of routine screening in HR-HPV-positive women has shown that the triage performance of self-sampling *MAL/miR-124-2* methylation for CIN2+ is non-inferior to cytology using physician-collected smears, with a shorter diagnosis time but higher referral rate (152). A methylation assay of *PAX1*, *SOX1*, and *ZNF582* has achieved moderate to high agreement between self-collected samples and physician-collected cervical scrapes (138). In a large prospective multicenter cohort study of matched self-collected cervicovaginal lavage samples and physician-taken cervical smears, a *FAM19A4* methylation assay has slightly lower sensitivity but higher specificity to detect CIN3+ in self-collected samples than physician-taken ones in the HR-HPV-positive gynecologic outpatient population. Moreover, addition of HPV16/18 genotyping to this methylation assay could achieve an almost identical sensitivity between these two types of samples. Therefore, the combination of *FAM19A4* methylation and HPV16/18 genotyping may represent a robust test with similar triage capacity in both sample types (44).

Unlike cytology that requires high sample quality, complicated screening procedures, well-trained/experienced cytologists, and resulting high rate of loss to follow-up (44, 153), a self-sampling approach is able to achieve one-sample one-visit screening, The latter remarkably simplifies the screening procedure and thus reduces the rate of loss to follow-up, especially in LMICs with high HR-HPV incidence but limited opportunity for screening (20, 154). However, several studies have shown that the self-sampling approach has a relatively high rate of colposcopy referral and thus an increase in medical expenses (152). Therefore, further investigations are required to address this issue to verify the value of the self-sampling DNA methylation assays in large prospective population-based screening trials.

## Conclusions and Perspectives

With a marked reduction in the incidence of CIN/ICC due to effective screening and treatment in developed countries, more than 80% of cervical cancer cases currently occur in LMICs (155), primarily because of lacking various resources necessary for screening (156, 157). In this case, a simple, quick, affordable test using an objective biomarker that can be performed in the same sample used for the primary screen *via* a single “screen and treat” visit may help overcome this challenge in LMIC (158).

The standard of care for the triage of HR-HPV-positive women mainly includes cytology, p16-INK4A/Ki-67 immunostaining, and HPV16/18 genotyping (159). Cytology is widely used as a triage approach in developed countries (11), while the results may vary due to the influence of sampling, storage, and result interpretation (127, 160). Thus, the cytological test requires well-preserved cell morphology, complicated procedures, and skilled pathologists, which is thus not suitable for self-sampling high-throughput screening (161). Notably, an approach of cervical cytology, named ThinPrep Cytologic Test (TCT), has been developed to detect cervical cells using a liquid-based thin-layer cell detection system. TCT may improve cytological screening of CINs, particularly increasing the positive diagnosis rate of the small number and small size of high-grade squamous epithelial lesions. Nevertheless, some HPV-positive women with negative cytology may have dormant CIN lesions, even cervical cancer (162). Cross-sectional and longitudinal studies have revealed that p16-INK4A or dual-p16-INK4A and Ki-67 immunostaining is an alternative triage approach for women with HR-HPV infection (15, 163). Similar to cytology, this approach is also not suitable for application in LMICs due to lack of medical resources. HPV16/18 genotyping is recommended in the referral guidelines of the United States (164). It has a specificity of approximately 80% but a low sensitivity (~60%) for detecting CIN2+, therefore increasing colposcopy referral (161, 165, 166). The high rate of colposcopy referral increases financial burden and thus prevents HPV16/18 genotyping in LMICs (167, 168). Although several studies suggest the use of *E6/E7* mRNA detection as a triage approach for HR-HPV-positive women (169, 170), its ability as an independent triage test has been considered insufficient (12, 171). In addition, HPV16/18 genotyping would miss other types of HPV infections.

Emerging evidence supports that DNA methylation changes in promoter regions of host genes and the HPV DNA genome often occur prior to carcinogenesis in ICC, which can be detected using the same sample of the HR-HPV test by relatively inexpensive assays (58, 116, 172–174). Thus, this kind of epigenetic abnormalities can be utilized either alone or in combination with cytology or HPV16/18 genotyping, for the triage of HR-HPV-positive women. These approaches are able to identify HR-HPV-infected patients that have a low short-term risk of development to ICC, especially young women with fertility requirements, who need only follow-up or treatment after childbirth. Compared to other triage approaches, the DNA methylation detection is mechanism-based and thus more precise, as well as more convenient with potential to be automated. Moreover, the methylation test can be performed on the same samples used for the HR-HPV test, thereby particularly suitable for self-collected samples, which simplifies the screening procedure and improves acceptability and coverage (11, 36, 175). This would particularly benefit LIMCs where the participation rate of screening is quite low (176). Moreover, the development of a high-throughput approach to improve the efficiency of DNA methylation detection could make it as a point-of-care test (same-day screening and treatment), which may significantly reduce the rate of loss to follow-up. Unlike cytology, the results of DNA methylation analysis are more homogeneous in multicenter studies (51, 152). Unlike HPV16/18 genotyping, DNA methylation detection is not restricted to the detection of CIN2+ involving HPV16/18 infection but also has higher sensitivity and comparable specificity for CIN2+ (20). More importantly, this type of triage approaches is anticipated to be advanced quickly due to the discovery of more sensitive and specific methylated genes and the development of new methodologies for genome-wide analysis, such as methylated DNA immunoprecipitation (MeDIP), methylated CpG island recovery assay combined with microarray analysis, and next-generation sequencing. They would allow the evaluation of novel CIN-specific methylation markers in population-based screening trials (53).

However, a number of issues remain to be addressed before applying DNA methylation detection for screening CIN/ICC. In most published studies, clinical samples for the methylation test were mainly obtained from patients screened by cytology or colposcopy referrals, which may not represent the women who anticipate population-based screening programs primarily using the HR-HPV test (51, 55). Moreover, as the indication for biopsy largely relies on the results of colposcopy, not all women enrolled into the published studies had histological endpoints, which could result in misclassification of some lesions due to the considerable variation in the sensitivity of cytology and colposcopy (177). Also considering the lower percentage of HPV-positive women with CIN lesions in the screening population than the gynecologic referral population, caution needs to be taken in translation of the findings from those studies into population-based screening (44). Cervical scraping is definitely more suitable for population-based screening and thus expected to be used widely in the near future. The value for identifying CIN3+ by a combination of multiple methylation markers in cervical tissue samples is different from that for cervical scrapes, probably due to the variation of background methylation levels caused by diverse cell types in tissue samples, but not scrapings. Therefore, the findings from the studies using tissue samples may not be applicable in cervical scrapes (68). For example, the gentle procedure, to avoid bleeding that causes poor visualization of colposcopy, for cervical scrape sampling may affect the quality of scrape samples (e.g., low concentrations of DNA that could lead to invalid results) (49). For example, a study has shown that the majority of DNA methylation results (~90%) obtained from cervical scrapes taken immediately before colposcopy could be invalid (44). Differences in the cutoff values used in the published studies may attribute to different screening population, sample size, specimen storage, etc. Thus, those cutoffs for DNA methylation tests need to be further validated in comparable populations, together with standardized procedures for sample collection and storage (122). A large proportion of the prospective studies have been conducted in the European populations (e.g., Netherlands and UK), of which most findings thus need to be further verified in other ethnic populations to avoid potential bias (20). Lastly, simple and inexpensive techniques for DNA methylation detection should be developed to make it more suitable for low-resource settings in LMICs. In this context, a consensus primer for amplifying 12 oncogenic HPV types has been developed to minimize the number of PCR reactions, representing a promising tool to detect viral methylation in women with HR-HPV infections (178).

Together, numerous studies have demonstrated that the DNA methylation detection of host cell genes or the HPV genome, or both, can serve as a primary approach for the triage of CIN/ICC. There are many advantages for using DNA methylation tests as a triage approach, including higher objectivity, more convenience, and comparable performance to cytology (158). This kind of triage approach could also help improve the allocation of healthcare resources to focus more on the treatment of high-risk women while sparing low-risk ones who only need conservative follow-up, especially in LMICs where the resources are quite limited. An ideal screening method requires both high sensitivity for detecting HR-HPV infections and strong capability for defining different infection statuses, together with adequate specificity and robust PPV for distinguishing CIN lesions (124). To this end, an increasing number of methylation markers have been shown to have higher sensitivity and non-inferior specificity compared to cytology. However, the majority of them have been investigated at the early stages in different populations thus far, while only a few have advanced to the late stage of clinical trials, and none has been approved for clinical use in daily practice. Thus, the DNA methylation markers and particularly their combination panels identified so far remain to be optimized (particularly by increasing triage specificity without impairing their high sensitivity), which may also require the identification of more novel methylation markers and panels. Nonetheless, with those advantages (e.g., relatively high sensitivity and non-inferior specificity compared to cytology, suitability for self-collected samples, and inexpensiveness), the host cell gene/HPV DNA methylation test represents a promising approach for the triage at the population level, allowing the early detection and accurate risk stratification of CINs to prevent their progression toward fatal ICC. Although there are several remaining concerns that need to be addressed, the introduction of host cell gene and HPV DNA methylation detection as a triage approach may lead to the era of molecular risk stratification for CIN lesions in HR-HPV-positive women, in order to precisely predict early and therefore prevent their progression to ICC in the near future.

## Author Contributions

LZ, SZ, and YD conceptualized, designed, and wrote the review. WT and HY contributed to the literature collection and data interpretation. All authors contributed to the article and approved the submitted version.

## Funding

This work was supported by the National Natural Science Foundation of China (grant numbers 81471165, 81670190, 81671108, 81670189, and 81870160), Natural Science Foundation of the Jilin Province (grant numbers 20190201042JC and 20190201163JC), Science and Technology Development Program of the Jilin Province (No. 20210509010RQ), and Interdisciplinary Integration and Innovation Project of JLU.

## Conflict of Interest

The authors declare that the research was conducted in the absence of any commercial or financial relationships that could be construed as a potential conflict of interest.

## Publisher’s Note

All claims expressed in this article are solely those of the authors and do not necessarily represent those of their affiliated organizations, or those of the publisher, the editors and the reviewers. Any product that may be evaluated in this article, or claim that may be made by its manufacturer, is not guaranteed or endorsed by the publisher.

## Abbreviations

HPV, human papillomavirus; HR-HPV, high-risk human papillomavirus, LMICs, low- and middle-income countries; CIN, cervical intraepithelial neoplasia; CIN1, grade 1 cervical intraepithelial neoplasia; CIN2, grade 2 cervical intraepithelial neoplasia; CIN3, grade 3 cervical intraepithelial neoplasia; CIN2+, CIN2 or worse; CIN3+, CIN3 or worse; ICC, invasive cervical cancer; SCC, squamous cell carcinoma; AdCA, adenocarcinoma; AIS, adenocarcinoma *in situ*; PPV, poor positive predictive value; NPV, negative predictive values; E2BS, E2-binding sites; URR, upstream regulatory region; DNMT, DNA methyltransferase; TP53, tumor protein p53; pRb, retinoblastoma protein; 5mC, 5-methylcytosine; CADM1, cell adhesion molecule 1; TSLC1, tumor suppressor in lung cancer 1; MAL, myelin and lymphocyte protein; CCNA1, cyclin A1; JAM3, junctional adhesion molecule 3; TERT, telomerase reverse transcriptase; ZSCAN1, zinc finger and SCAN domain containing 1; ST6GALNAC5, ST6 N-acetylgalactosaminide alpha-2,6-sialyltransferase 5; ANKRD18CP, ankyrin repeat domain 18C; PAX1, paired box gene 1; PAX2, paired box gene 2; CDH1, cadherin 1; CDH6, cadherin 6; GFRA1, GDNF family receptor alpha 1; GATA4, GATA-binding protein 4; KCNIP4, potassium voltage-gated channel interacting protein 4; LHX8, LIM homeobox 8; SOX1, SRY-box transcription factor 1; SOX17, SRY-box transcription factor 17; ZNF582, zinc finger protein 582; ZNF671, zinc finger protein 671; PTPRR, protein tyrosine phosphatase receptor type R; POU4F3, POU class 4 homeobox 3; DAPK1, death-associated protein kinase 1; RARB, retinoic acid receptor beta; ASTN1, astrotactin 1; DLX1, distal-less homeobox 1; ITGA4, integrin subunit alpha 4; RXFP3, relaxin family peptide receptor 3; CALCA, calcitonin-related polypeptide alpha; ESR1, estrogen receptor 1; APC, adenomatous polyposis coli; RRAD, Ras-related glycolysis inhibitor and calcium channel regulator; SFRP1, secreted frizzled related protein 1; MT1G, metallothionein 1G; NMES1, normal mucosa of esophagus-specific gene 1; SPARC, secreted protein acidic and cysteine rich; TFPI2, tissue factor pathway inhibitor 2; ADCYAP1, adenylate cyclase activating polypeptide 1; ASCL1, achaete-scute family BHLH transcription factor 1; DCC, deleted in colorectal carcinoma; DBC1, deleted in bladder cancer protein 1; HS3ST2, heparan sulfate-glucosamine 3-sulfotransferase 2; MYOD1, myogenic differentiation 1; TMEFF2, transmembrane protein with EGF like and two follistatin-like domains 2; TIMP3, tissue inhibitor of metalloproteinases 3; RASSF1, Ras association domain family member 1; PRDM14, PR/SET domain 14; qMSP, quantitative methylation-specific PCR; MeDIP, methylated DNA immunoprecipitation; AUC, area under the curve

## Glossary

HPV: human papillomavirus
HR-HPV: high-risk human papillomavirus
LMICs: low- and middle-income countries
CIN: cervical intraepithelial neoplasia
CIN1: grade 1 cervical intraepithelial neoplasia
CIN2: grade 2 cervical intraepithelial neoplasia
CIN3: grade 3 cervical intraepithelial neoplasia
CIN2+: CIN2 or worse
CIN3+: CIN3 or worse
ICC: invasive cervical cancer
SCC: squamous cell carcinoma
AdCA: adenocarcinoma
AIS: adenocarcinoma *in situ*
PPV: poor positive predictive value
NPV: negative predictive values
E2BS: E2-binding sites
URR: upstream regulatory region
DNMT: DNA methyltransferase
TP53: tumor protein p53
pRb: retinoblastoma protein
5mC: 5-methylcytosine
CADM1: cell adhesion molecule 1
TSLC1: tumor suppressor in lung cancer 1
MAL: myelin and lymphocyte protein
CCNA1: cyclin A1
JAM3: junctional adhesion molecule 3
TERT: telomerase reverse transcriptase
ZSCAN1: zinc finger and SCAN domain containing 1
ST6GALNAC5: ST6 N-acetylgalactosaminide alpha-2,6-sialyltransferase 5
ANKRD18CP: ankyrin repeat domain 18C
PAX1: paired box gene 1
PAX2: paired box gene 2
CDH1: cadherin 1
CDH6: cadherin 6
GFRA1: GDNF family receptor alpha 1
GATA4: GATA-binding protein 4
KCNIP4: potassium voltage-gated channel interacting protein 4
LHX8: LIM homeobox 8
SOX1: SRY-box transcription factor 1
SOX17: SRY-box transcription factor 17
ZNF582: zinc finger protein 582
ZNF671: zinc finger protein 671
PTPRR: protein tyrosine phosphatase receptor type R
POU4F3: POU class 4 homeobox 3
DAPK1: death-associated protein kinase 1
RARB: retinoic acid receptor beta
ASTN1: astrotactin 1
DLX1: distal-less homeobox 1
ITGA4: integrin subunit alpha 4
RXFP3: relaxin family peptide receptor 3
CALCA: calcitonin-related polypeptide alpha
ESR1: estrogen receptor 1
APC: adenomatous polyposis coli
RRAD: Ras-related glycolysis inhibitor and calcium channel regulator
SFRP1: secreted frizzled related protein 1
MT1G: metallothionein 1G
NMES1: normal mucosa of esophagus-specific gene 1
SPARC: secreted protein acidic and cysteine rich
TFPI2: tissue factor pathway inhibitor 2
ADCYAP1: adenylate cyclase activating polypeptide 1
ASCL1: achaete-scute family BHLH transcription factor 1
DCC: deleted in colorectal carcinoma
DBC1: deleted in bladder cancer protein 1
HS3ST2: heparan sulfate-glucosamine 3-sulfotransferase 2
MYOD1: myogenic differentiation 1
TMEFF2: transmembrane protein with EGF like and two follistatin-like domains 2
TIMP3: tissue inhibitor of metalloproteinases 3
RASSF1: Ras association domain family member 1
PRDM14: PR/SET domain 14
qMSP: quantitative methylation-specific PCR
MeDIP: methylated DNA immunoprecipitation
AUC: area under the curve
